# Real-Time Prediction of the Yarn Break Position Using Vibration Measurement

**DOI:** 10.3390/s25020299

**Published:** 2025-01-07

**Authors:** Marcin Idzik, Tomasz Rybicki

**Affiliations:** 1 Institute of Automatic Control, Lodz University of Technology, 90-537 Lodz, Poland; tomasz.rybicki@p.lodz.pl; 2 Lukasiewicz Research Network—Lodz Institute of Technology, 90-570 Lodz, Poland

**Keywords:** warping machine, yarn, detection, MEMS

## Abstract

Warping is a crucial process that connects two main stages of production: yarn manufacturing and fabric creation. Two interrelated parameters affect the efficiency of this technological process: warping speed and the ability to swiftly detect the yarn breaks caused by various defects. The faster a break is detected and the warping machine stopped, the higher the machine’s working speed can be. Since the beginning of such devices, various types of yarn break detectors have been proposed, primarily based on different mechanical solutions. To enhance the break detection process, a solution involving the use of an accelerometer to measure yarn vibrations and thereby detect whether the moving yarn has broken is proposed. Based on the detection of a threshold value of 22 m/s^2^, the warping machine could be stopped within 2.752 to 2.808 ms, which is 50 times faster than in the traditional mechanical detectors under investigation. Furthermore, through a precise analysis of yarn vibration patterns, it became possible to determine the distance from the sensor at which the break occurred. This analysis was conducted using the proprietary MRSCEK coefficient, which aggregates data obtained from six standard coefficients: mean, root mean square, standard deviation, crest factor, energy, and kurtosis. This information could potentially lead to the development of automated systems for removing breaks without human intervention in the future. Research efforts focused on analyzing the vibration signals received from yarns made with different linear densities. The results showed that such a system could effectively replace commonly used mechanical yarn break detectors and operate much faster.

## 1. Introduction

The yarn break detection system described in this work is a highly significant component of warping machines. It is responsible for sending timely information to the machine about breaks and the immediate need to stop. If the system did not detect the break, the end of the yarn would be wound onto the warp beam and covered by subsequent layers of yarns that were being wound simultaneously, parallel to each other. In such a scenario, identifying a defect would be extremely difficult. Due to the crucial role played by the yarn break detection system in the production process, it must possess not only high speed but also absolute reliability and relatively low installation costs. Consequently, dozens of diverse solutions for detecting breaks have been developed to date. Despite the passage of time, this issue remains relevant and continues to be a subject of interest [1,2,3,4].

Despite the continuous development of newer prototypes, the most widely used system for yarn break detection, combining simplicity, reliability, cost-effectiveness, and speed, remains the “dropper”. Its construction relies on a dropper, which is suspended from the yarn that passes through it. When a break occurs, the tension in the yarn vanishes, causing the dropper to fall downward (sometimes assisted by a spring) and make contact with two rods, thus closing an electrical circuit. This action sends a signal to the machine about the yarn break and the immediate need to halt operations [5].

Given the necessity of examining hundreds of yarns simultaneously, many studies have explored the potential use of cameras employing image analysis techniques to detect emerging yarn breaks. These solutions are based on CCD (charge-coupled devices) or CIS (contact-type image sensor) cameras. Their setup involves moving the yarn over a special table with a contrasting color to the yarn and illuminated by dedicated light sources, with a camera capturing the yarn’s movement. When a break occurs, the yarn disappears from the camera’s field of view, generating an appropriate signal [6,7]. Many solutions focused on analyzing very narrow spatial segments [8,9]. An example includes a device that examines enlarged images of viscose yarn to detect broken fibers protruding from the yarn [10]. Devices were also designed to analyze images of already woven fabric, identifying broken yarns, which constitute defects in the final product [11]. The latest and most advanced project involves real-time yarn break detection by analyzing the entire frame of the warping machine [12].

For many years, a widely researched method for yarn break detection has involved using light-sensitive elements. Initially, the focus was on employing sensors to detect light from a series of diodes placed beneath the moving yarns, which would be blocked by the yarn under normal conditions [13,14]. However, yarn vibrations and variations in warp production from different types of yarn significantly compromised the universality of such solutions. With the development of lasers, these were also examined as yarn break detectors. Their operational logic closely resembled the previously described method. The laser beam from an emitter was meant to be blocked by the yarn before reaching the receiver [15,16]. In this case, substantial vibrations from the moving yarn and its differing linear mass proved to be significant limitations, requiring the precise calibration of the system each time. Another approach involved illuminating the yarn with laser light and capturing the reflected beam with a detector [17,18].

Summing up the above examples, despite significant technological advancement, a completely new yarn break detection system based on modern electronic systems rather than classical mechanical detection setups has not been developed. By leveraging the phenomenon of vibrations, which has been problematic and minimized until now, it seems possible to design a system capable not only of detecting the occurrence of a yarn break but also of determining how far from the detector the break occurred. Implementing such a solution could streamline machine operation and yield information that might enable full automation of the yarn break removal process, which is currently performed manually.

The introduction of measurements using MEMS (microelectromechanical system) sensors into the textile industry was initiated within the scope of detecting yarn breakage in Jacquard weaving. The system operated by recording the movement of the harness and checking for any changes in the behavior of the analyzed element. This enabled the detection of the moment when the yarn broke, leading to alterations in the operation characteristics of the harness [19,20,21].

This article presents the concept of yarn break detection using an accelerometer. This research involved designing the measurement system and analyzing the received signals. The results exhibit better performance parameters than traditional yarn break detectors, indicating promising prospects for the widespread adoption of this developed solution in the future. Additionally, the proposed measurement system provides information that was previously unavailable from mechanical yarn break detectors—the precise location of the break. It opens up wide possibilities for utilizing such information in future systems aiming to fully automate the warping process.

This work is divided into three main sections. Section 2 and Section 3, “Materials” and “Methods”, the components used and their arrangement in the project are presented, along with a brief description of the idea behind the project. Section 4, titled “Results and Discussion”, contains a detailed description of the conducted research and its discussion. Lastly, Section 5, titled “Conclusions”, summarizes all the studies and work carried out during the execution of this interdisciplinary project.

## 2. Materials

### 2.1. Warping Machine

The research was conducted using a direct warping machine of the DS. 14P type from Karl Mayer Textilmaschinenfabrik GmbH, Obertshausen, Germany [22]. This machine allows for the production of warps from both natural and synthetic yarns. The warp width is adjustable within a range of 8 to 15 inches. The machine is equipped with a “magazine” frame that accommodates dual yarn packages, enabling the creation of warps that are twice as long. Being a direct warping machine, it is intended for producing long, uniform warps, and equipping it with a yarn compensator significantly increases the warping speed. The type of the studied object does not affect the final results; however, implementing the system with a different machine will require its initial calibration.

### 2.2. Yarns

During this research, yarns made from various materials were used, encompassing both natural fibers like cotton and synthetic ones such as polyester and viscose. These examined yarns also varied in their linear mass, ranging from 20 to 50 tex.

### 2.3. Tension Measurement

An electronic strain gauge from Rothschild-Instruments, type R-1092, was used to measure the yarn tension.

### 2.4. Hairiness Measurement

The Uster Tester 3, a universal device used to measure yarn parameters, was employed to measure the hairiness coefficient. This device enables the measurement of various yarn parameters, including the Uster evenness value CVm, imperfections, hairiness, hairiness length classification diameter, density, shape, twist, dust and trash, and yarn fineness.

### 2.5. Software

During the construction of the research setup, three software tools were utilized. The first was Arduino IDE ver. 2.3.0, which was used to write the algorithm for collecting data from the accelerometer. Additionally, the use of PuTTY ver. 0.81 software was necessary to gather data from the serial port and save it in a comma-separated values (.csv) file. Finally, the acquired data underwent analysis using MATLAB R2023b software.

### 2.6. Hardware

The designed measurement system consisted of three devices. The primary component was the Arduino Uno R3 microcontroller, which was responsible for retrieving specific data from the sensor. The next device was the SparkFun DEV-14495 converter (https://www.sparkfun.com/products/14495 (accessed on 3 January 2025)), facilitating the connection of signal wires from the microcontroller and, on the other end, the QWIIC standard cable from the accelerometer. The use of this adapter was necessary to minimize the influence of the wires on the sensor’s movement as much as possible. The final and most crucial element of the system was the 3-axis accelerometer and gyroscope, LSM6DSO32 6DoF IMU, from Adafruit (https://www.adafruit.com/product/4692 (accessed on 3 January 2025)). It allows for measuring acceleration up to ±32 g, with a maximum measurement frequency of 6.66 kHz. For the purposes of this work, to increase accuracy, the measurement range was reduced to ±8 g and the maximum reading speed was set to 6.66 kHz.

## 3. Methods

### 3.1. The Idea of Measuring Yarn Vibration

Despite significant technological advancements, the operation of looms remains relatively unautomated. Major tasks causing machines to spend more time in preparation than in actual operation involve linking old warps with new ones (to avoid threading the yarn throughout the entire machine). Depending on the type of machine, these linking tasks can range from several hundred to several thousand. Furthermore, despite thorough machine preparation, yarn breaks occur during operation, leading to machine stoppage and the need to locate and manually remove such defects [23,24,25].

To reduce the time needed to locate yarn breaks and expedite the machine’s response to such breaks, a concept for a detector has been developed. This detector, utilizing yarn vibration measurements, aims not only to identify the occurrence of a yarn break but also to calculate the precise location along the several-meters-long frame where the break occurred.

### 3.2. Measuring System

**Figure 1:**
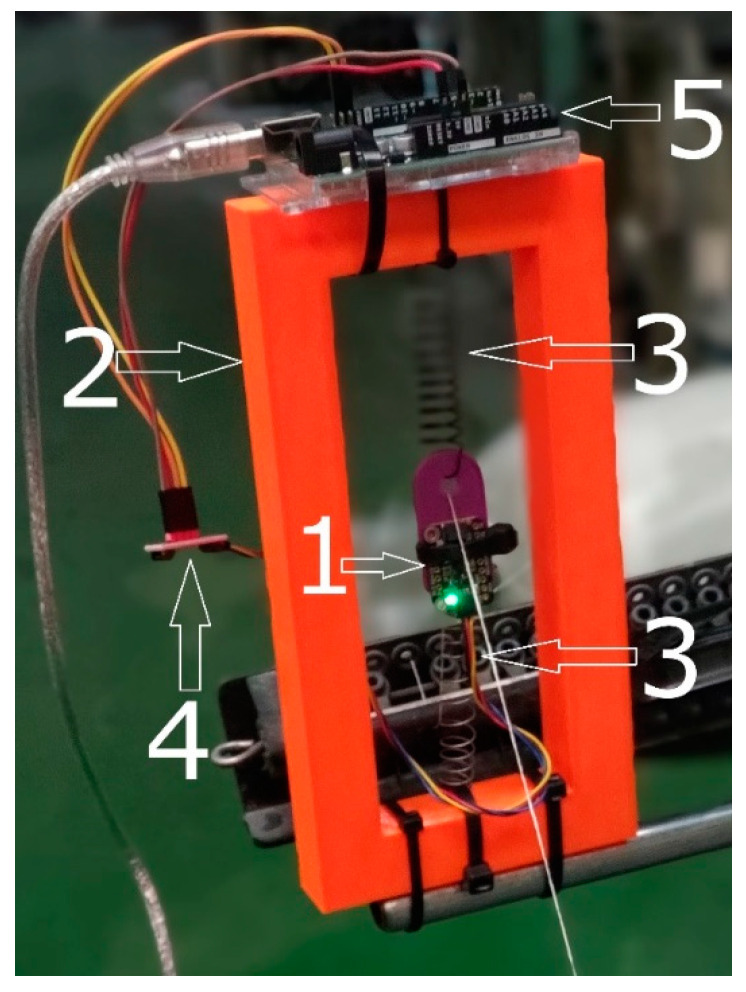
Measuring system (1—accelerometer, 2—supporting frame, 3—isolating springs, 4—signal converter, 5—microcontroller).

In Figure 1, a schematic of the measurement system is depicted, comprising: 1—accelerometer Adafruit LSM6DSO32 6DoF IMU, 2—supporting frame made from PCL material with a 3D printer, 3—isolating springs to insulate the sensor from solid surfaces and external vibrations, 4—signal converter SparkFun DEV-14495, and 5—microcontroller Arduino Uno R3. The principle of operation of the detector involves its deviation from its initial position due to tensioned yarn passing beneath it. When a yarn break occurs, the tension in the yarn vanishes, causing the sensor to return to its initial position.

### 3.3. Production of Yarn

One of the primary characteristics indicating the quality of yarn is the frequency and size of the naturally occurring slubs and thickened areas within it. In commonly available yarns, these defects are not only minimized (understandably) but also their exact location within the yarn is typically unknown. Consequently, for the fundamental purpose of inducing yarn breaks in very specific locations as part of the described research, it was necessary to produce special yarn using available industrial-laboratory machines.

The yarns intended for this research were made from staple cotton, viscose, and polyester fibers. A medium-spindle spinning system of cotton and cotton-like fibers was utilized. Spinning slivers were produced on a carding machine separately for each raw material. During the carding process, impurities, short fibers, and neps (tangled fiber fragments) were removed from the fibrous mass. The neps were collected for subsequent yarn modeling. To even out the linear mass distribution and parallelize the fibers, the slivers were joined (6 splices in total) and drafted in the drafting rollers of two consecutive passages in the stretching machines.

All the carding and stretching slivers had a uniform linear mass of 4 ktex.

From the slivers after the second stretching, rovings were produced using a ring spinning frame. A stretch ratio of 10 was applied in the drafting units of the spinning frame, allowing for the production of rovings with a linear mass of 400 tex. These rovings were given a twist of 50 turns per meter, forming cylindrical–conical packages.

The process of forming yarn from the rovings was carried out on a classic ring spinning frame. Various drafting ratios were employed in the machine, resulting in yarns with different linear masses. Yarns with linear masses ranging from 20 tex to 50 tex were produced on the ring spinning frame. The thicker yarns used in the research were obtained by joining and twisting finer yarns on a laboratory double-twisting machine [26].

The technological process aimed to produce yarns with the most even distribution of linear mass. However, for research purposes, yarns with a specific model structure were also needed, which were characterized by a distinct distribution of slubs and thickened areas in the form of neps. This model structure was obtained directly on the ring spinning frame by periodically removing some fibers locally from the feeding roving, resulting in the formation of slubs in the created yarn. Additionally, neps were added to the feeding roving at specific intervals; these had previously been removed during the carding process. This addition allowed them to twist into the yarn, creating characteristic thickened areas. During the experiments on the tensile tester, these thickened areas led to momentary increases in yarn tension and yarn breaks at the slub locations.

### 3.4. Theoretical Assumptions

The project assumes that it is possible to detect yarn breakage and determine its location by measuring vibrations using a sensor placed at the end of the warping machine frame. This assumption stems from the fact that, during machine operation, the sensor experiences a tension force (*S*), which is the sum of two friction forces. The first force is generated in the tensioner (*T*), while the second force is the friction force (*F*) acting on the elements supporting the yarn along its entire path from the bobbin to the warp beam. The combined action of these forces causes the extension of the spring where the sensor is positioned.

(1) S=T+F

During a yarn break, the force system undergoes a sudden change. The friction force related to the tensioner disappears because it is the first element in the yarn’s path, and the break always occurs before it. However, the friction force is reduced according to Formula (2):

(2) T=μ·m·g

where *μ*—friction coefficient, *g*—gravitational acceleration, and *m*—yarn mass.

Since the coefficient of friction remains constant, the only change lies in the mass acting on the frictional surfaces. This mass depends on a crucial parameter—the location of the yarn break and, hence, the length and weight of the remaining yarn.

In summary, during a break, a force acts on the sensor that is equal to the difference between the forces present before and after the event, according to Formula (3). Therefore, it can be inferred that the acceleration measured by the sensor will be constant for a specific break point and will vary depending on the location of the break along the warping machine frame:

(3) $$X=-(T+\mu \cdot g\left(m_{2}-m_{1}\right))$$

where *m*_1_—mass of the yarn before the break, and *m*_2_—mass of the yarn after the break.

To estimate the possibility of detecting changes in sensor acceleration concerning the location of yarn breaks, calculations were performed based on measurements of the technological parameters during machine operation. This research was conducted for three linear masses of yarn: 20, 50, and 100 tex, assuming that there are three fixed break points located at distances of 1, 2, and 3 m from the sensor. The results of these measurements are presented in Table 1. For each yarn type, two measurements were taken. The first was conducted during normal machine operation, while the second omitted the tensioning system. This allowed the subtraction of the two values to determine the force acting on the sensor during a break. Additionally, for each measuring point, the weight of the remaining yarn after the break was determined and added to the sensor’s own mass, which is 4.52 g. This process enabled the calculation of the acceleration that would be measured by the sensor in the actual setup.

**Figure 2:**
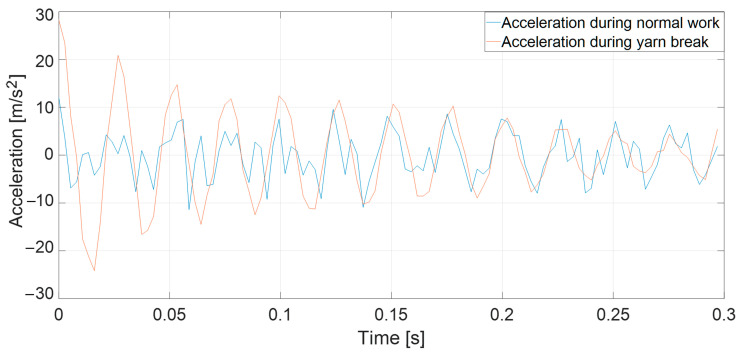
Plot showing an example of sensor acceleration along the vertical *x*-axis over time during normal warping machine operation and during breakage of the yarn.

Based on the obtained results, it can be observed that the measured accelerations do not significantly differ depending on the break location. Additionally, the yarn is not a uniform object—it has various surface defects that influence the generated tension force [27,28,29]. Moreover, there is the effect of variable friction force and the formation of balloons during the unwinding of the yarn from the bobbins [3,30]. Confirmation of this hypothesis is presented in Figure 2, depicting the acceleration as measured by the sensor along the vertical *x*-axis during the warping machine’s operation. These described fluctuations cause significant variations in the sensor’s acceleration pattern, consequently leading to an inability to extract the desired data.

**Figure 3:**
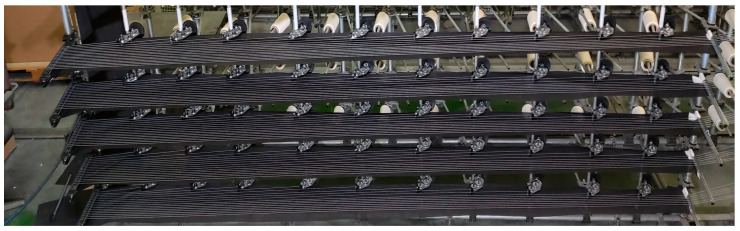
Non-woven layer under the yarns.

The problem described above was addressed by adding a layer of nonwoven fabric just beneath the moving yarn along the warping machine’s frame. Its purpose is to significantly increase the frictional force when the yarn breaks and falls onto it. This action leads to a several-fold increase in the difference in frictional force between consecutive measurement points. The research findings are presented in Table 2; Figure 3 shows the nonwoven fabric under the yarns in the warping machine.

The problem described above was addressed by adding a layer of nonwoven fabric just beneath the moving yarn along the warping machine’s frame, which is depicted in Figure 3.

**Figure 4:**
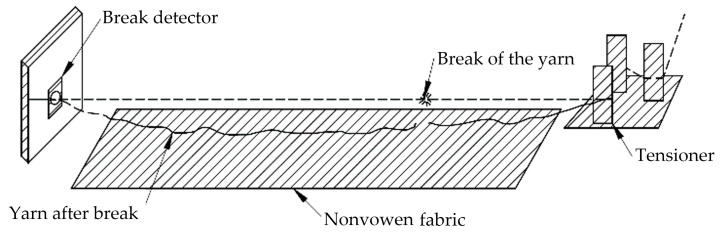
Conceptual diagram.

Hydro-needled nonwoven fabric weighing 35 g/m^2^ was used, which was immersed in a solution of water and resin with the addition of soot. Then, the excess solution was squeezed out between pressure rollers, and the prepared nonwoven material was subjected to a drying and stabilization process at a temperature of 150 °C. This resulted in a relatively stiff material in a black color—contrasting with the white yarns. Its purpose is to significantly increase the frictional force when the yarn breaks and falls onto it. This action leads to a several-fold increase in the difference in frictional force between consecutive measurement points. The research findings are presented in Table 2 and Figure 4 shows a conceptual diagram of the presented assumption.

Thanks to the nonwoven fabric pad, the differences between individual measurement points became significantly greater, allowing for a reduction in the impact of yarn tension fluctuations during operation on the ability to detect breakage.

Due to the described yarn defects, the measured signal at the moment of breakage does not consistently exhibit the same initial amplitude. Therefore, a straightforward comparison of amplitudes and the calculation of the breakage location using Table 2 is not possible. To achieve the set goal, it was decided that the system would halt the warping machine when the acceleration exceeded the threshold value of 22 m/s². Subsequently, the obtained signal was examined from the moment of stoppage to 0.3 s after it. This measurement time is necessary because the high-speed warping machine must not allow the broken yarn from the tensioners closest to the frame’s end to pass through the detector. This broken yarn causes an acceleration spike, disrupting comparisons with other yarns.

Based on an analysis of the nature of the measured vibration, six coefficients were selected, which were determined from the obtained vibration signal. They are described below.

1 Mean

The *Mean* (4) of the analyzed signal indicates its degree of symmetry. If it tends toward zero, the measured vibrations are symmetrical [31]:

(4) $$Mean=\frac{1}{N}\sum_{n-1}^{N}x(n)$$

where *x*(*n*) is the value of a particular sample *n*, and *N* is the number of all *n* samples.

2 Root Mean Square (*RMS*)

The root mean square (5) is a measure of the power contained in the signal [32].

(5) $$RMS\left(x\left(n\right)\right)=\sqrt{\frac{1}{N}\sum_{n-1}^{N}{x(n)}^{2}}$$

3 Standard Deviation (*SD*)

The standard deviation (6) is a coefficient indicating how much the measured signal deviates from its mean [32].

(6) $$SD\left(x\left(n\right)\right)=\sqrt{\frac{1}{N}\sum_{n-1}^{N}{\left[x\left(n\right)-mean(x(n))\right]}^{2}}T=\mu \cdot m\cdot g$$

4 Crest Factor

The *crest* factor (7) determines the ratio of the peak values of the signal to its root mean square value. For a sinusoidal wave, this is equal to 1.414 [32].

(7) $$Crest\left(x\left(n\right)\right)=\frac{max\;|x\left(n\right)|}{RMS(x\left(n\right))}$$

5 Energy

The *energy* (8) of the signal is measured by summing the square of all its values [31].

(8) $$Energy\left(x\left(n\right)\right)=\sum_{n-1}^{N}{\left(x(n)\right)}^{2}$$

6 Kurtosis Coefficient

*Kurtosis* (9) is a coefficient designed to detect minor defects in vibrations [33].

(9) $$Kurtosis\left(x\left(n\right)\right)=\frac{\frac{1}{N}\sum_{n-1}^{N}{\left(x\left(n\right)-mean(x\left(n\right))\right)}^{4}}{{\left[\frac{1}{N}\sum_{n-1}^{N}{\left(x\left(n\right)-mean(x\left(n\right))\right)}^{2}\right]}^{2}}-3$$

## 4. Results and Discussion

### 4.1. Detection of Yarn Breaks with Different Tex Values

**Figure 5:**
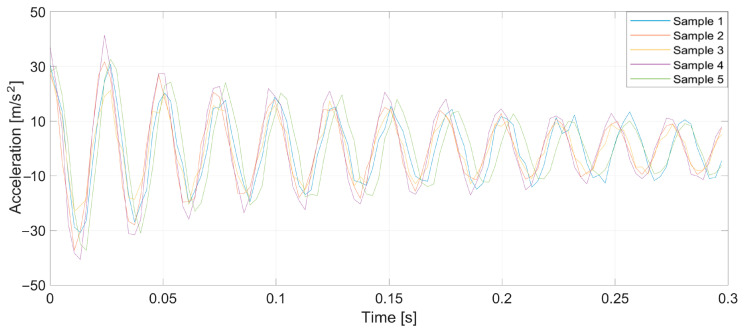
Plot of sensor acceleration over time: yarn, 20 tex; break, 1 m.

**Figure 6:**
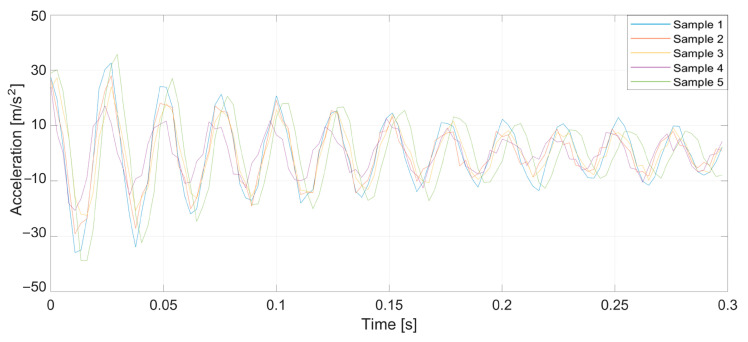
Plot of sensor acceleration over time: yarn, 20 tex; break, 2 m.

**Figure 7:**
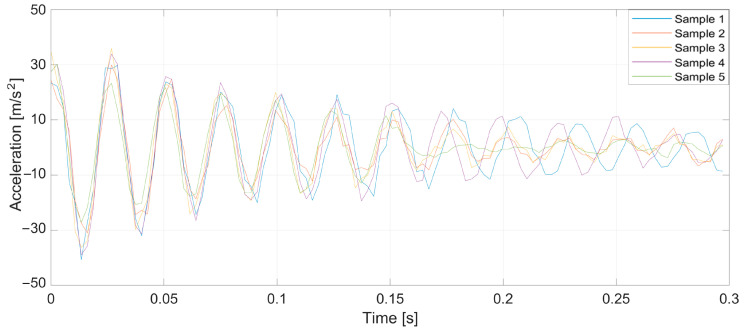
Plot of sensor acceleration over time: yarn, 20 tex; break, 3 m.

**Figure 8:**
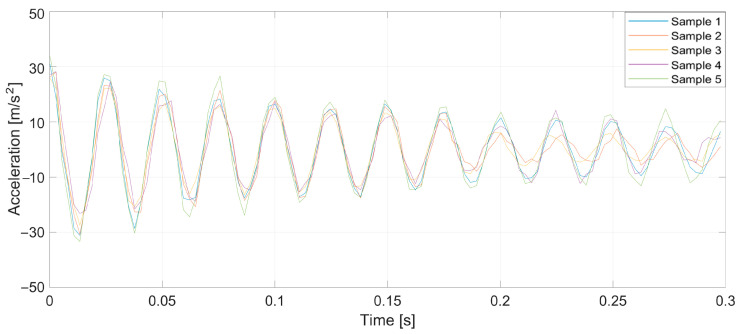
Plot of sensor acceleration over time: yarn, 50 tex; break, 1 m.

**Figure 9:**
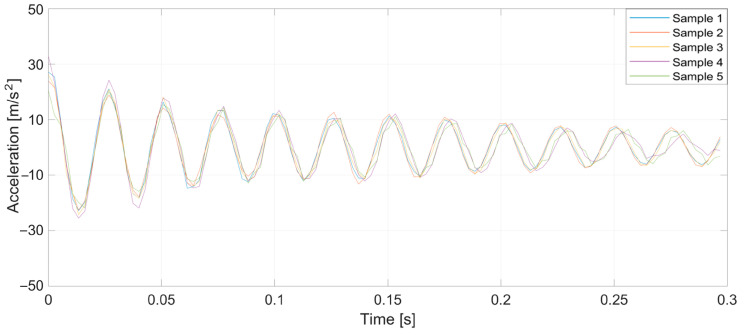
Plot of sensor acceleration over time: yarn, 50 tex; break, 2 m.

**Figure 10:**
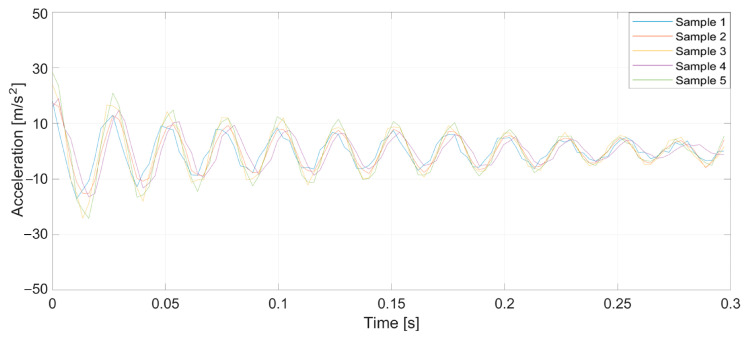
Plot of sensor acceleration over time: yarn, 50 tex; break, 3 m.

To verify the validity of the method, which was drawn up from the earlier theoretical considerations, a series of tests was conducted. Specifically, prepared yarn was used as the warp in the normal cycle of the warping machine’s operation. Introducing a pair of defects into the yarn caused a significant increase in tension when a large thickened area passed through the tensioners, leading to breakage at the intentionally created weak point. Three representative breaking points were selected, each at distances of 1, 2, and 3 m from the tensioner. At this stage of the research, yarns of 20 and 50 tex (grams per kilometer of yarn) were used, and five measurements were taken at each point. Figure 5, Figure 6, Figure 7, Figure 8, Figure 9 and Figure 10 depict the accelerations in the vertical *x*-axis that were registered at each measurement point.

A summary of the obtained results is presented in Table 3 and Table 4, from which the coefficients described in the previous section were calculated for yarn breaks with linear masses of 20 and 50 tex.

Comparing the results shown in Table 3 and Table 4, it can be seen that the only parameter allowing us (although not in every case) to determine the location of the yarn break is the value of the calculated vibration energy. Nevertheless, these values are not very precise, and some results coincide with those obtained at a different break location. However, it was noticed that with greater vibration energy, the value of the RMS and SD coefficients also increased. To obtain more valuable data from the calculated coefficients and based on the observed dependencies between the coefficients, a special Formula (10) was developed, calculating the MRSCEK coefficient (Table 5). It sums up four coefficients: mean, root mean square, standard deviation, and crest factor. Then, the calculated sum is squared and divided by the products of energy and kurtosis. The results obtained in this way are presented in Table 4.

(10) $$MRSCEK=\frac{{\left(Mean+RMS+SD+Crest\right)}^{2}}{Energy\cdot Kurtosis}$$

**Figure 11:**
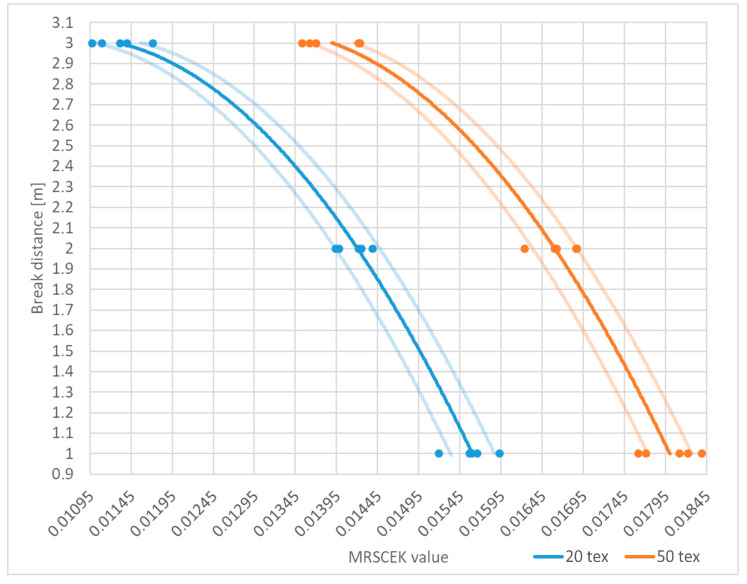
Plot of MRSCEK values (the average is represented by solid lines and the expanded uncertainty is represented by transparent solid lines; measuring points are marked with dots).

Summarizing the above, it can be seen that the average measurement results for a break occurring 2 m from the sensor compared to a break occurring 1 m from the sensor are smaller by 0.00144 for 20 tex yarn and 0.00138 for 50 tex yarn. Between the breaks at 3 and 2 m, the difference is 0.00285 and 0.00278, respectively. Therefore, they are approximately twice as large. Consequently, it can be stated that by determining the coefficients described in the article and substituting them into the proposed formula, it is possible to determine with a certain accuracy the location along the warping machine frame where the yarn break occurred. Furthermore, the application of the proposed formula minimizes the impact of linear mass on the final measurement result. Figure 11 portrays the results of MRSCEK coefficient calculations for all trials and for the average value.

To make it easier to read the position of the yarn break, based on the average values from the measurements, the course of the distance values relative to the MRSCEK coefficient was approximated using a quadratic polynomial. The course of this polynomial is shown as solid lines in Figure 11. For a yarn of 20 tex linear weight, the function has the form f_20_(x) = −85920x^2^ + 1846.1x − 6.8903, while for 50 tex, f_50_(x) = −83882x^2^ + 2188x − 11.207, where x is the value of the MRSCEK factor. As the measurements taken were not identical, an expanded uncertainty value was determined for each measurement point with a coverage factor of 2 and, therefore, for a confidence level of 95%. Conservatively, the highest value of the expanded uncertainty of 0.000269 was assumed for all measurements, which is presented in Figure 11 as continuous semi-transparent lines above and below the main function. From this figure, the approximate yarn break distances can be easily read off, based on the calculated MRSCEK coefficient. For example, for a 20 tex yarn and an MRSCEK coefficient value of 0.01345, the break occurred at a distance of 2.3966 m (+0.1189/−0.1313).

### 4.2. Detection of Yarn Breaks with Different Materials

Another fundamental variable during warping yarn is the material from which it was made. For the study, yarns made from three types of fibers, namely, cotton, polyester, and viscose, were used. All of them were produced using the same method, as described in the previous section for cotton yarns. To verify if the material used for yarn-making affects the obtained results, a series of tension measurements were conducted. The results proved inconclusive because no noticeable change in tension was observed for the different material types. Dynamic measurements exhibit fluctuations in tension, stemming not only from the variable frictional forces generated while unwinding yarn from bobbins (cross-winding) but also from fluctuations in the friction forces associated with the non-uniformity of the yarn along its length.

To precisely examine the impact of material on the obtained results, the hairiness coefficient H [29,34] was measured using the Uster Tester 3 laboratory device. All yarns had the same linear mass of 50 tex. The measurement results are presented in Table 6.

Hairiness is one of the key factors that can affect the coefficient of friction of yarns. Based on the obtained results, it can be stated that regardless of the material, the hairiness coefficient is very similar, and its fluctuations are so minor that they do not significantly impact the acceleration measured by the sensor and, thus, the ability to determine the location of yarn breakage. The differences between yarn samples made from the same material are greater than the differences between values for different materials.

### 4.3. Algorithm Running Time

The stopping speed of the machine after detecting a broken yarn is a critical parameter for any warping machine. This duration is influenced by two primary factors. The first is the efficiency of the braking system, which is responsible for stopping the rotating warp beam. The second is the activation speed of the yarn breakage detection system. Table 7 presents the measurements of the descent speed of mechanical detectors, conducted in four different warping machines.

In the designed measurement system, successive samples are collected every 1 ms. Since only information about the occurrence of yarn breakage is required to stop the warping machine, while the precise localization of the breakage can be determined later, it is necessary to check if the measured acceleration value has exceeded a certain threshold. Executing these actions and generating the yarn status signal takes between 2752 and 2808 ms, enabling the detection of yarn breakage about 50 times faster than with classical mechanical solutions.

## 5. Conclusions

Based on our research, a new method for measuring yarn breakage using the acceleration signals obtained from an accelerometer has been designed and tested. This method allows us not only to determine the occurrence of yarn breakage but, more importantly, to identify the location where the breakage occurred. Key to achieving these results was the development of the proprietary MRSCEK coefficient, which proved to be more effective than analyzing individual coefficients when examining the recorded signal. It takes into account six coefficients: mean, root mean square, standard deviation, crest factor, energy, and kurtosis. As demonstrated in the conducted studies, the MRSCEK coefficient is primarily dependent on the distance of the yarn breakage from the accelerometer. The second significant parameter is the linear mass of the yarn, in which an increase causes a parallel shift of the MRSCEK values upward.

The parameters obtained from the proposed method are naturally dependent on the warping machine (friction of the underlying fabric, braking distance, operating speed, etc.) and the type and mass of the yarn. However, they can be easily determined and recorded in tables referenced by the system for analyzing the location of the breakage.

An important aspect of the research was the development of a method for producing yarn with the desired properties, with particular emphasis on defects being placed at designated locations. This was precisely the opposite approach to most studies conducted on the yarn-processing process, which focuses on obtaining products with the best, most flawless parameters.

As the study of yarn hairiness showed, hairiness is very similar regardless of the type of material from which the yarn was made. The H values of all samples ranged from 4.58 to 4.65. However, in future studies, it is worth analyzing in detail the influence of the degree of yarn hairiness on the friction coefficient and comparing it with the values obtained from the MRSCEK coefficient calculations.

It is also worth noting that the developed measurement system allows for very fast measurements. As the research showed, classical mechanical detectors achieve operating times ranging from 0.116 to 0.124 s, translating into obtaining a reaction time over 50 times faster by the proposed accelerometer-based solution. By achieving operating speeds ranging from 2.752 to 2.808 ms, it can be concluded that the designed system operates in real time and drastically shortens the braking distance of the warping machine.

The implementation of this project opens up wide possibilities for the development of robotic systems allowing for the automatic tying of broken yarns. So far, there have been no systems in common use that allow for determining the exact location of yarn breakage; thus, there has been a lack of the necessary data for automating the process of removing breakages.

## Figures and Tables

**Table 1:** Acceleration of the yarn during breakage.

Linear Mass [tex]	20			50		
Tension during work [cN]	31			31		
Tension without tensioner [cN]	17			17		
Difference in forces after a break [cN]	14			14		
Break distance from sensor [m]	1	2	3	1	2	3
Mass of the sensor with yarn [g]	4.56	4.58	4.60	4.63	4.68	4.73
Acceleration [m/s^2^]	30.70	30.56	30.43	30.23	29.91	29.60

**Table 2:** Acceleration of the yarn during breakage with the increase in friction force.

Linear Mass [tex]	20			50		
Tension during work [cN]	31			31		
Tension without tensioner [cN]	17	18	19	17.5	19	21
Difference in forces after a break [cN]	14	13	12	13.5	12	10
Break distance from sensor [m]	1	2	3	1	2	3
Mass of the sensor with yarn [g]	4.56	4.58	4.60	4.63	4.68	4.73
Acceleration [m/s^2^]	30.48	28.38	26.09	29.16	25.64	21.14

**Table 3:** Values of coefficients for the performed measurement of 20 tex yarn.

Break [m]	Probe	Mean	RMS	SD	Crest	Energy	Kurtosis
1	1	−0.1150	12.8432	12.3591	2.1958	18,509	2.5680
1	2	−0.1924	13.2199	12.6775	2.8192	19,399	2.7608
1	3	0.0418	10.6006	10.2035	2.5593	12,773	2.6911
1	4	−0.1147	15.9945	15.3479	2.5853	28,096	2.6142
1	5	−0.0161	14.2478	13.9217	2.6137	22,233	2.7306
2	1	−0.3014	13.6565	13.1104	2.6288	20,701	2.8685
2	2	−0.1053	11.0380	10.5852	2.6427	13,524	3.0994
2	3	0.2063	10.1475	9.6638	2.6641	11,956	2.9912
2	4	−0.1040	9.2037	8.8075	2.9227	9603	3.1783
2	5	−0.0576	14.7197	13.9899	2.8750	22,101	3.2164
3	1	−0.1821	13.0912	12.6884	3.0356	20,605	3.3977
3	2	0.0180	10.7358	10.3783	2.9424	11,321	4.6655
3	3	−0.0163	12.7506	12.1491	2.8430	16,546	4.1061
3	4	0.0829	14.0465	13.4887	2.7354	22,729	3.5541
3	5	0.1892	10.3428	9.8647	2.9209	11,874	4.1275

**Table 4:** Values of coefficients for the performed measurement of 50 tex yarn.

Break [m]	Probe	Mean	RMS	SD	Creast	Energy	Kurtosis
1	1	−0.1709	123857	11.8999	2.5182	16,028	2.4413
1	2	−0.0969	11.5041	11.0278	2.6721	13,690	2.6120
1	3	0.0982	10.1163	9.6865	2.7164	11,360	2.5425
1	4	0.2168	10.9402	10.5168	2.5767	12,285	2.6263
1	5	−0.1098	14.2205	13.6876	2.3747	19,447	2.5456
2	1	0.0853	9.4016	8.9916	2.8761	9611	2.8575
2	2	0.0279	9.0800	8.7188	2.6266	9152	2.7106
2	3	−0.0431	9.4056	9.0049	2.7739	9820	2.7399
2	4	0.0587	9.8296	9.2512	2.6321	9990	2.7038
2	5	−0.0944	8.3405	8.0238	2.6210	7722	2.8460
3	1	−0.0341	6.2277	5.9646	2.7731	4905	3.1923
3	2	−0.0629	8.1979	7.8247	2.9386	7460	3.3687
3	3	0.0245	8.5824	8.2675	2.9188	8176	3.4965
3	4	0.1461	6.1142	5.8526	3.0879	4750	3.5695
3	5	0.0432	8.8920	8.4823	3.1770	8277	3.7876

**Table 5:** The results of the MRSCEK coefficient calculations.

Break [m]	Probe	20 tex	50 tex
1	1	0.0157	0.0181
1	2	0.0152	0.0176
1	3	0.0159	0.0177
1	4	0.0156	0.0182
1	5	0.0156	0.0184
	Mean	0.0156	0.0180
2	1	0.0143	0.0166
2	2	0.0139	0.0169
2	3	0.0144	0.0166
2	4	0.0142	0.0169
2	5	0.0140	0.0162
	Mean	0.0142	0.0166
3	1	0.0117	0.0142
3	2	0.0110	0.0142
3	3	0.0113	0.0137
3	4	0.0114	0.0136
3	5	0.0111	0.0135
	Mean	0.0113	0.0139

**Table 6:** Testing the hairiness of yarn.

Material	Cotton	Polyester	Viscose
Sample 1	4.58	4.62	4.58
Sample 2	4.61	4.64	4.65
Sample 3	4.65	4.59	4.60

**Table 7:** Mechanical break detector activation times in seconds.

**The Name of the Machine**	**Sample 1**	**Sample 2**	**Sample 3**
KARL MAYER DS.14P	0.123	0.123	0.124
HUYS & VANHEVEL NV type Sigma	0.116	0.115	0.115
LIBA type 23	0.121	0.121	0.121
ELITEX type 2206	0.119	0.119	0.118

## Data Availability

The data used to support the findings of this study are available from the author upon request.
